# Splenectomy or Partial Splenectomy should be Preferred Treatment for Large Splenic Hydatid Cysts

**DOI:** 10.4269/ajtmh.16-0112

**Published:** 2016-06-01

**Authors:** Abhimanyu Sharma

**Affiliations:** Department of Pathology University college of Medical Sciences Delhi, India Guru Tegh Bahadur Hospital Delhi, India E-mail: abhimanyu20s@gmail.com

Dear Sir:

The article by Arce and others was helpful.^1^ However, in cases of giant splenic hydatid cyst, splenectomy or partial splenectomy is generally considered the preferred treatment rather than removal of the cyst, as reported by Rashid and others.^2^ If there is more than one cyst, a large cyst, a cyst in a deep organ location, or an infected cyst, open surgery should be done.^2^ I agree with Arce and others that the laparoscopic approach is the preferred treatment for splenic hydatid disease, but only in the case of a small cyst. Also, removal only of the cyst is not the recommended treatment in the literature for splenic hydatidosis, especially for large cysts. Similar recommendations have been made by Eris and others.^3^ I agree that preservation of the spleen whenever possible is justified because of its role in protecting against infection.^2^ But in the case of large hydatid cysts in an elderly patient, splenectomy or partial splenectomy should be preferred.
